# Cadaveric training model for the endovascular management of type B aortic dissection

**DOI:** 10.1016/j.jvscit.2025.101916

**Published:** 2025-07-11

**Authors:** Peter Osztrogonacz, Daanish Sheikh, Dylan Brooks, Bahar Alasti, Paul Haddad, Rebecca Barnes, Stuart J. Corr, Alan B. Lumsden, Kamal Safadi, Robert Burns, Maham Rahimi

**Affiliations:** a Department of Cardiovascular Surgery, Houston Methodist Hospital, Houston, TX; b Department of Vascular and Endovascular Surgery, Semmelweis University, Budapest, Hungary; c Long School of Medicine, UT Health San Antonio, San Antonio, TX; d Department of Kinesiology, McMaster University, Hamilton, Ontario, Canada; e Department of Bioengineering, Rice University, Houston, TX; f Swansea University Medical School, Institute of Life Science 2, Swansea, United Kingdom

**Keywords:** Atherosclerosis, Endovascular, Renal transplant

## Abstract

**Background:**

The surgical management of type-B aortic dissection (TBAD) poses considerable technical challenges, necessitating meticulous planning and precise execution. In an effort to enhance the proficiency of trainees in the management of TBAD through thoracic aortic endovascular repair, we have developed a cadaveric TBAD training model.

**Methods:**

We conducted a feasibility test using a plastic tube designed to simulate the basic anatomical characteristics of the aorta. To access the interior of the tube, we introduced a 26 Fr and a 5 Fr sheath at each end. Employing a soft glidewire, we fashioned a proximal loop around the proximal segment of a Dacron graft (DG). Subsequently, a distal loop was created at the distal end of the DG using a glidewire. The DG was then carefully maneuvered through the 26 Fr sheath within the simulated “aorta” by traction on the distal end of the proximal loop, which extended outward from the 5 Fr sheath. Finally, visualization of the DG within the “aorta” was achieved using an intravascular ultrasound catheter. This methodology was subsequently replicated in a cadaveric model, as detailed in the following section.

**Results:**

The in vitro feasibility test substantiated the viability of the devised concept for TBAD model creation. Encouraged by these findings, we proceeded to establish a cadaveric TBAD model. Access was gained to the left common carotid and right common femoral arteries, facilitating the placement of an undersized DG distal to the left subclavian artery, employing the previously described methodology. Completion angiography verified the successful creation of the TBAD model. In the conclusive phase, a Gore cTAG endograft was deployed distal to the left subclavian artery.

**Conclusions:**

The presented model not only demonstrated the feasibility of our conceptual approach for TBAD training model creation but also underscored the potential reproducibility of a cadaveric TBAD model. This innovative educational tool holds promise for effectively instructing vascular trainees in the intricate nuances of surgical management for TBAD.

Aortic dissection is the most prevalent pathology within the acute aortic syndrome spectrum.^1^ Type B aortic dissection (TBAD) presents a technically demanding pathology for endovascular treatment, necessitating thorough preparation of trainees for operative management. Currently, a well-established training model catering to the requisite skillset for addressing this challenging aortic pathology is lacking. In response to this gap and drawing upon our prior expertise with cadaveric models,^2^^,^^3^ we have developed a cadaveric TBAD model to enhance trainee skillsets and proficiency in TBAD management through thoracic aortic endovascular repair (TEVAR).

## Methods

**Fig 1:**
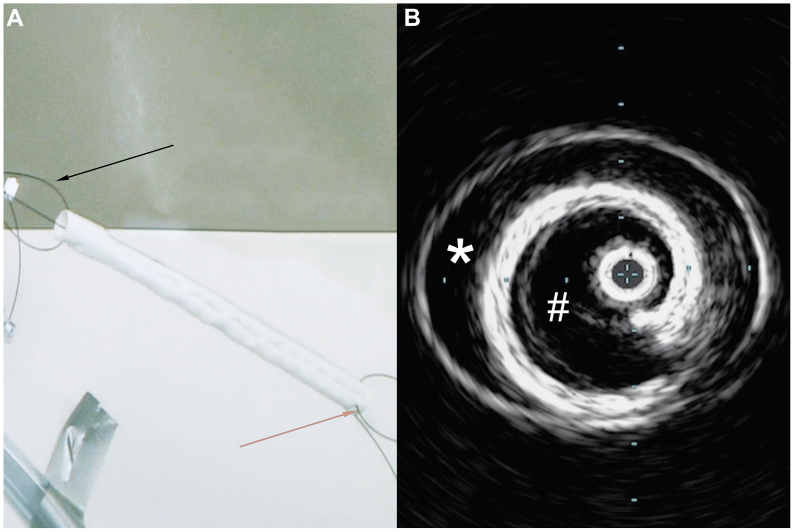
**(A)** Photograph of the Dacron graft (DG) device used to simulate a type B aortic dissection (TBAD) in a cadaver. The *black arrow* points to the “proximal” loop and the *orange arrow* towards the “distal” loop. **(B)** Intravascular ultrasound (IVUS) was performed within the DG in an in vitro model to visualize the graft in relation to the “aortic” wall.

**Fig 2:**
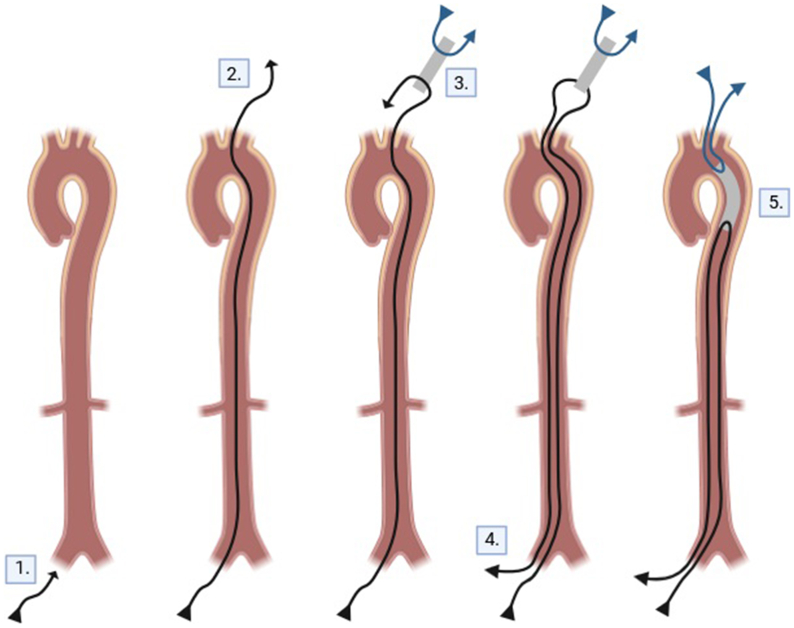
Schematic depicting the construction of the in vivo type B aortic dissection (TBAD) model within a cadaver.

Initiating with an in vitro feasibility test using a plastic tube closed at both ends to simulate the aorta, we accessed the “aorta” through a 26 Fr sheath and a smaller 5 Fr sheath. A soft glidewire, introduced through the smaller sheath, was snared and externalized through the larger bore sheath. This end of the glidewire was threaded through the lumen walls of a Dacron graft (DG) creating a “proximal” loop (Fig 1, *A*, *black arrow*). A second glidewire was threaded through the walls of the opposing “distal” end to form a “distal” loop (Fig 1, *A*, *orange arrow*). Subsequently, the free end of the initial glidewire of the proximal loop was fed back to the 26 Fr sheath and externalized through the 5 Fr sheath such that the proximal loop could be used to draw traction from the 5 Fr sheath, whereas the distal loop could be used to draw traction on the DG from the 26 Fr sheath. The DG was then pulled into the simulated aorta through the large bore sheath. The simulated aorta was filled with saline, and an intravascular ultrasound (IVUS) catheter was inserted within the graft for visualization in relation to the “aortic” wall (Fig 1, *B*). A schematic depicting the insertion process in vivo is depicted in Fig 2.

**Fig 3:**
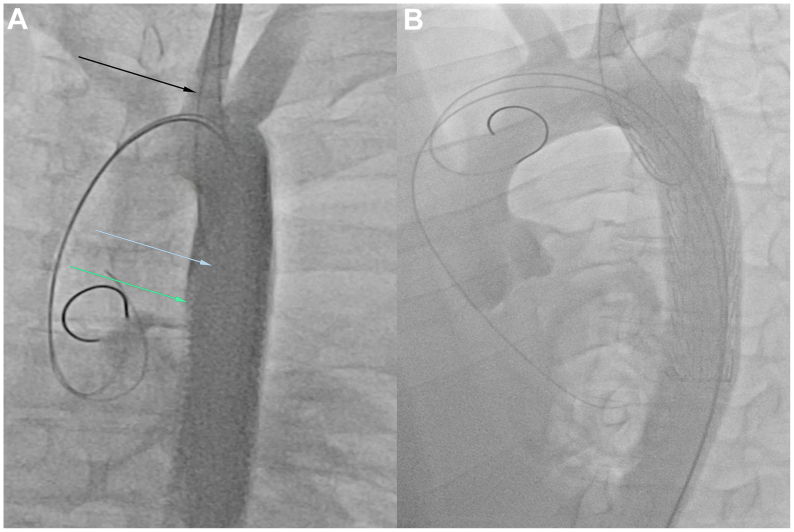
**(A)** Fluoroscopy depicting the constructed in vivo type B aortic dissection (TBAD) model. The *blue arrow* points towards the Dacron graft (DG) representing the true lumen, the *green arrow* points towards the ”false“ lumen, and the *black arrow* points towards the proximal glidewire loop for graft positioning. **(B)** Fluoroscopy depicting the cadaveric TBAD model following repair with a deployed stent graft.

**Fig 4:**
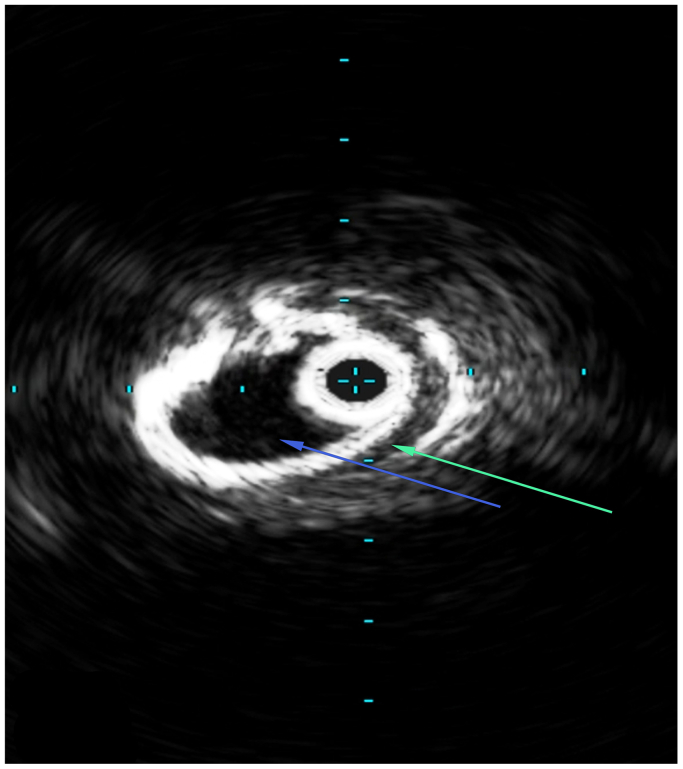
Intravenous ultrasound (IVUS) depicting the cadaveric aorta prior to repair. The *blue arrow* indicates the true lumen and the *green arrow* indicates the false lumen.

The cadaveric TBAD model creation, conducted at the state-of-the-art training facility of Houston Methodist Hospital (MITIE), utilized our experience with the in vitro TBAD model. A fresh frozen cadaver served as the model. Access was gained to the left common carotid artery (CCA) and bilateral common femoral arteries (CFA), with sheaths placed appropriately. A glidewire, introduced through the left CCA, was snared and externalized through the right CFA. The proximal end of a 10-cm DG was punctured, and the glidewire was driven through the DG fabric hole from the femoral site. The glidewire was then fed back into the femoral sheath and externalized through the CCA access site using a snare. A super-stiff Amplatz wire was introduced into the aorta through the DG lumen up to the aortic root. The distal end of the DG was secured with a second glidewire, facilitating graft introduction into the aorta by pulling on the first glidewire. The position of the DG was adjusted using the proximal and distal glidewire loops, ultimately placing it distally from the left subclavian artery origin in the zone 3 segment (Fig 3, *A*). Subsequently, the CCA and left CFA sheaths were connected to an arthroscopic irrigation pump, generating pulsatile flow with body temperature saline. Finally, a Gore CTAG stent graft (W. L. Gore & Associates) was deployed via the super-stiff Amplatz wire to the zone 3 segment of the thoracic descending aorta (Fig 3, *B*). Cone-beam computed tomography (CBCT) and IVUS confirmed aortic dissection-like morphology with distinct true and false lumens prior to repair (Fig 4).

**Fig 5:**
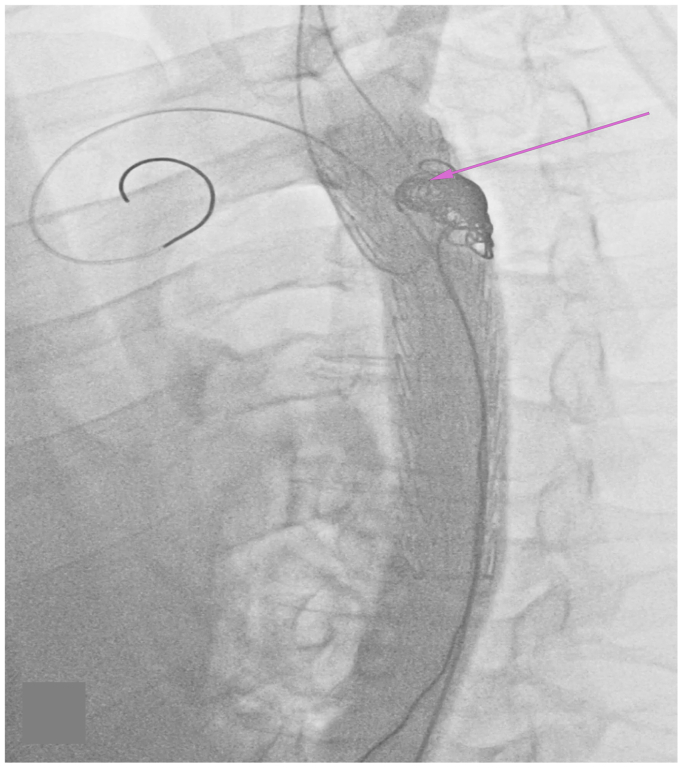
Fluoroscopy depicting the cadaveric aorta during the candy-plug repair. The stent graft within the true lumen is visible. The *pink arrow* depicts the interlock coils used to embolize the proximal aspect of the false lumen.

**Fig 6:**
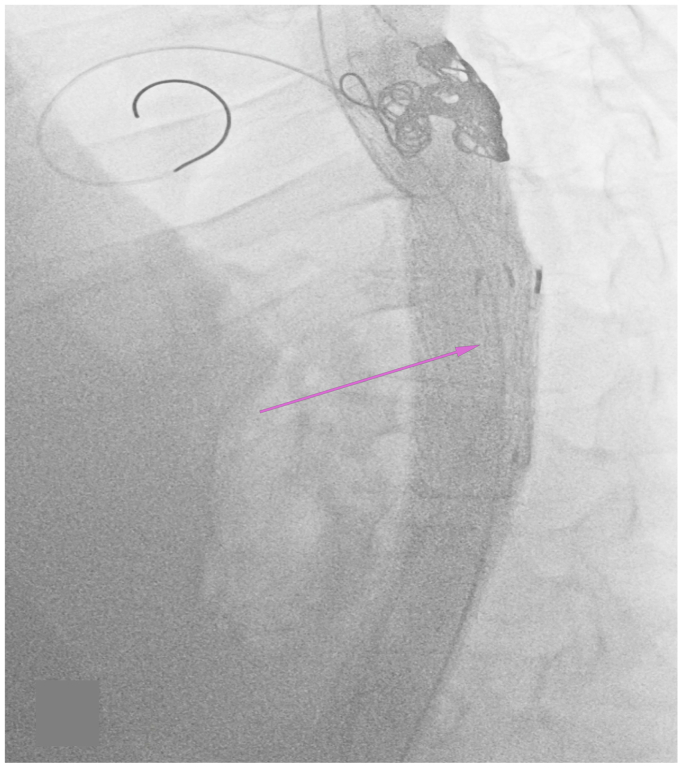
Fluoroscopy of the cadaveric aorta following candy-plug deployment. The *pink arrow* depicts the candy-plug occluding the distal aspect of the false lumen.

A vascular surgeon also demonstrated the feasibility of performing more complex aortic dissection repair techniques in this cadaveric model. To perform the PETTICOAT and STABILISE techniques, a 26 mm × 10 cm Gore CTAG thoracic stent graft (W. L. Gore & Associates) was first deployed just distal to the left subclavian artery to cover the primary entry tear. Next, Cook dissection stents (Cook Medical), overlapping the already placed stent graft, were extended down to the aortic bifurcation to realign the dissected lumen and complete the PETTICOAT technique. To demonstrate the STABILISE technique, a Coda balloon catheter was then used to postdilate the bare metal stents, intentionally rupturing the lamella to restore true lumen integrity. To simulate the tactile feedback of lamellar rupture, the model’s DG or “true lumen” was cut longitudinally and sewn back together using 6-0 Prolene suture, such that dilating the balloon would need to overcome suture tension. Finally, the superior mesenteric artery and both renal arteries were selectively cannulated and stented to maintain perfusion to visceral aortic branches. To demonstrate the candy-plug technique, a 26 mm × 10 cm Gore CTAG stent graft (W. L. Gore & Associates) was first introduced via the right groin over a Lunderquist wire (Cook Medical) and deployed just distal to the left subclavian artery. Interlock coils were then used to embolize the proximal aspect of the false lumen, initiating thrombosis (Fig 5). A custom candy plug was fashioned from an aortic cuff, which was secured with a silk tie to create the characteristic “plug” configuration (Supplementary Video 1, online only). This plug was then positioned within the false lumen to promote durable occlusion and prevent distal re-entry flow (Fig 6).

## Discussion

Although existing aortic dissection models primarily involve animal models^4^, ^5^, ^6^ and vascular phantoms,^7^^,^^8^ both approaches present significant drawbacks. Animal models are time-consuming with a relatively high failure rate, whereas vascular phantoms lack appropriate feedback on endovascular devices and do not facilitate the practice of steps involved in real-life cases, such as ultrasound-guided access. In contrast, our cadaveric TBAD model offers a lifelike experience, mimicking the conditions trainees will encounter in the operating room. The model provides feedback on endovascular devices, enables ultrasound-guided access, and allows for planning using CBCT and intraoperative decision-making based on IVUS. Moreover, although a more realistic practice setting for standard aortic dissections is helpful for trainees, the real value of the cadaveric model lies in teaching less frequently utilized and complex techniques, such as STABILISE and candy-plug, which address persistent false lumen filling. Because these procedures are used only in selected cases, trainees might not otherwise obtain appropriate exposure to these kinds of techniques to become proficient.

A male cadaver with a normal body mass index was chosen to ensure a generally larger aortic diameter. Overcoming the challenge of creating aortic dissection on the native aorta in a cadaveric setting, our technique utilizing a DG addresses the significant issue of reproducibility.

The selection of the appropriate DG size is critical, necessitating a diameter small enough to separate false and true lumens in relation to the cadaveric aorta. Although a preoperative CBCT could determine the cadaver’s thoracic aorta diameter, its time-consuming nature led us to adopt a 12-mm DG. Considering the descending thoracic aorta’s diameter of 24.8 ± 3.49 mm, this DG size should be safely accommodated by most cadaveric aortas.

The proximal and distal loops created by regular glidewires facilitate optimal DG positioning inside the aorta. In our trials with the cadaveric model, our institution’s faculty observed that introducing pulsatile flow not only contributed to a lifelike ultrasound-guided access to the CFA but also enabled contrast clearance, mitigating issues of contrast accumulation and its impact on visibility with fluoroscopy.

Limitations include the cost and availability of cadavers, along with the infrastructure required for creating a cadaveric TBAD model and performing TEVAR.

Within the context of a potential TBAD workshop for trainees, our facility and cadaveric model provide an opportunity for preoperative CBCT, treatment planning, ultrasound-guided access of the CFA, IVUS to distinguish between true and false lumens, and TEVAR implantation in a lifelike environment. Regional centers equipped with the necessary infrastructure and access to cadavers could optimize spending and accommodate a large number of trainees to gain proficiency in TBAD management.

## Conclusions

Our proof-of-concept experiment suggests the feasibility of a lifelike and potentially reproducible cadaveric TBAD model. This innovative model has the potential to assist vascular trainees in gaining experience and proficiency in TBAD management within a safe and realistic environment.

## Funding

Funding was provided by the 10.13039/100015581Houston Methodist Research Institute (HMRI). HMRI had no influence on the study design, manuscript writing, decision to publish, or creation of the manuscript.

## Disclosures

A.L. receives research support from W.L. Gore & Associates; is a consultant at W.L. Gore & Associates, Boston Scientific, and Siemens; is a stock stakeholder at Hatch Medical; is a speaker at Boston Scientific, W.L. Gore & Associates, and Siemens; and is on the advisory board of Boston Scientific and AneuMed.

## Appendix (online only)

Supplementary Video Construction of the “candy plug” used in the candy-plug technique to seal the distal aspect of the false lumen.
